# Advances of Modern Chromatographic and Electrophoretic Methods in Separation and Analysis of Flavonoids

**DOI:** 10.3390/molecules13102521

**Published:** 2008-10-16

**Authors:** E-Hu Liu, Lian-Wen Qi, Jun Cao, Ping Li, Chang-Yin Li, Yong-Bo Peng

**Affiliations:** Key Laboratory of Modern Chinese Medicines (China Pharmaceutical University), Ministry of Education; Nanjing 210009, P.R. China; E-mails: lehabc@sina.com (E-H. L.), fleude@126.com (L-W. Q.), caojun91@163.com (J. C.), changyinli2006@126.com (C-Y. L.), ybpeng2008@126.com (Y-B. P.)

**Keywords:** Flavonoids, Sample preparation, Separation, Analysis, Gas chromatography, Liquid chromatography, Capillary electrophoresis

## Abstract

Flavonoids, one of the largest groups of secondary metabolites, are widespread in vegetable crops such as herbs, fruits, vegetables, grains, seeds and derived foods such as juices, wines, oils, *etc*. They receive considerable attention due to their biological and physiological importance. Hundreds of publications on the analysis of flavonoids have appeared over the past decade. Traditional and more advanced techniques have come to prominence for sample preparation, separation, detection, and identification. This review intends to provide an updated, concise overview on the recent development and trends of separation, identification and quantification for flavonoids by modern chromatographic and spectrophotometric analytical techniques, including gas chromatography (GC), liquid chromatography (LC), and capillary electrophoresis (CE). The sample preparation before analysis is also briefly summarized.

## 1. Introduction

Flavonoids are a group of polyphenolic compounds of low molecular weight that present a common benzo-γ-pyrone structure. They are categorized into various subclasses including flavones, flavonols, flavanones, isoflavanones, anthocyanidins, and catechins. Flavonoids are often hydroxylated in positions 3, 5, 7, 3′, 4′ and/or 5′. Frequently, one or more of these hydroxyl groups are methylated, acetylated, prenylated or sulphated. In plants, flavonoids are often present as *O*- or *C-* glycosides; *O* bonding occurs in flavonoids far more frequently than *C* bonding. The *O*-glycosides have sugar substituents bound to a hydroxyl group of the aglycone, usually located at positions 3 or 7, whereas the *C*-glycosides have sugar groups bound to a carbon of the aglycone, usually 6-C or 8-C. The most common carbohydrates are rhamnose, glucose, galactose and arabinose. Flavonoid-diglycosides are also frequently found. Two very common disaccharides contain glucose and rhamnose, 1→6 linked in neohesperidose and 1→2 linked in rutinose. The sugars are often further substituted by acyl residues such as malonate and acetate [1]. The average human diet contains a considerable amount of flavonoids, the major dietary sources of which include fruits (i.e. orange, grapefruit, apple, and strawberry), vegetables (i.e. onion, broccoli, green pepper, and tomato), soybeans and different herbs. 

Flavonoids in general have being studied for more than 70 years in *in vivo* and *in vitro* systems. They receive considerable attention in the literature, specifically because flavonoids are of biological and physiological importance. Plant flavonoids are involved in response mechanisms against stress, as caused by elevated UV-B radiation, infection by microorganisms or herbivore attack. Flavonoids are also involved in the production of root nodules as a nitrogen fixation system after infection by *Rhizobium* bacteria in a variety of leguminous plants. They have been shown to modify eicosanoid biosynthesis (antiprostanoid and anti-inflammatory responses), protect low-density lipoprotein from oxidation (prevent atherosclerotic plaque formation), prevent platelet aggregation (antithrombic effects), and promote relaxation of cardiovascular smooth muscle (antihypertensive, antiarrhythmic effects). In addition, flavonoids have been also shown to have antiviral, cytotoxic, anti-ulcerogenic, and carcinostatic activities [2].

The number of papers dealing with the assay of flavonoids in various analytical matrices is increasing in accordance with growing interest in the investigation of their pharmacological and biological effects. The present review focused on the current use of modern chromatographic and electrophoretic analytical techniques for the analysis of a wide variety of flavonoids – aglycones as well as conjugates – in many different sample types. The sample preparation techniques before analysis are also outlined. In all instances, selected real-life applications will be included to illustrate the practicability, and the scope and limitations of the various approaches. 

## 2. Sample preparation techniques for flavonoid analysis

### 2.1 General

Isolation of flavonoids from the sample matrix is generally a prerequisite to any comprehensive analysis scheme, although enhanced selectivity in the subsequent quantification step may reduce the need for sample manipulation. Over the years many sample pre-treatment methods have been developed to determine flavonoids in various sample types. There are three main types of flavonoid-containing matrices: plants, food and liquid samples such as biological fluids and drinks. The solid samples are usually first homogenized, which may be preceded by (freeze-) drying or freezing with liquid nitrogen. The next step is analyte isolation. For this purpose, solvent extraction (SE) – which may be followed by solid-phase extraction (SPE) – is still the most widely used technique, mainly because of its ease of use and wide-ranging applicability. Liquid samples are usually first filtered and/or centrifuged, after which the sample is either directly injected into the separation system or, more often, the analytes are isolated using liquid–liquid extraction (LLE) or SPE. 

Although many traditional sample-preparation methods for flavonoids are still in use, there have been trends in recent years towards: (1) use of smaller initial sample sizes, small volumes or no organic solvents; (2) greater specificity or greater selectivity in extraction; (3) higher recoveries or better reproducibility; and, (4) increased potential for automation. Recently, sample-preparation methods have been significantly improved. Sample treatment by means of pressurized liquid extraction (PLE), microwave-assisted extraction (MAE), supercritical-fluid extraction (SFE), SPE, matrix solid-phase dispersion (MSPD) and solid-phase micro-extraction (SPME) deserve more attention. 

### 2.2 Pressurized liquid extraction

Pressurized liquid extraction (PLE), known as accelerated solvent extraction, is a recently adopted extraction method that uses a high pressure and temperature with a forced flow of solvent. Compressed nitrogen is usually used as a carrier to move the solvent under high pressure. Increased temperature and elevated pressure accelerate the extraction kinetics so as to enable rapid extraction. Although PLE uses the same aqueous and organic solvents as traditional extraction methods, it makes more efficient use of them.

Recently, many papers on applying PLE to flavonoids analysis have been published. In Jiang’s [3] study, PLE was applied for five flavonoids extraction from *Lysimachia clethroide*. The extraction of flavonoids from *Houttuynia cordata* Thunb by PLE has also been reported [4]. The effects of several important factors such as the concentration of slovent, solvent rate, temperature and pressure were investigated by orthogonal array design experiment. Both a high flavonoids yield of 3.152% and a high flavoniods content of 23.962% were obtained with solvent ethanol concentration of 50%, solvent rate of 1.8 mL/min, temperature of 70 °C and pressure of 8MPa.

### 2.3 Microwave-assisted extraction (MAE)

Microwave-assisted extraction (MAE) involves heating solid sample-solvent mixtures in a closed vessel with microwave energy under temperature-controlled and pressure-controlled conditions. This closed extraction system enables analyte extraction with elevated temperatures and pressure accelerating the extraction process and yielding a performance comparable to the standard Soxhlet method. Besides the advantage of a high extraction speed, MAE also enables a significant reduction in the consumption of organic solvent (typically less than 40 mL, compared with the 100–500 mL required for Soxhlet extraction). Recently, MAE was employed for the extraction of flavonoids from *Herba Epimedii* [5]. The method has been compared with other extraction methods, such as pressurized microwave-assisted extraction (PMAE), ultrasonic extraction (UE), heat reflux extraction (RE) and Soxhlet extraction (SOX). The results showed that the extraction yield of flavonoids in *Herba Epimedii* obtained by dynamic microwave-assisted extraction (DMAE) was higher than that obtained by UE, RE and SOX, and the extraction time is dramatically reduced.

### 2.4 Solid-phase extraction

Solid-phase extraction (SPE) is one of the simplest, yet most effective and versatile, methods of sample preparation. It is widely used for flavonoid extraction and enrichment from aqueous samples and sample extracts. Utilizing low cost, prepacked, disposable cartridges, sample components of interest are separated from other species by applying the sample mixture to an appropriate chosen solid sorbent and selectively eluting the desired components. It is known that the efficiency of SPE depends on the type of sorbents, the sample volume and pH, the content of organic modifier and the volume of elution solvent. SPE cartridges that have been evaluated include: SH, GP, C_18_, C_8_, C_2_ and Oasis MCX mixed mode cartridges. In most cases the sorbent is C_18_-bonded silica and the sample solution and solvents are usually slightly acidified to prevent ionization of the flavonoids, which would reduce their retention. In the bioanalysis of flavonoids, solid phase extraction is a frequently used technique for sample pretreatment. A recent application is the simultaneous quantification of three isoflavonoid glycosides in rabbit plasma after oral administration of *Astragalus mongholicus* extract using LC–ESI–MS/MS [6]. The plasma with known amount standards and internal standard were extracted in a SPE column as follows: 300 μL volume of the plasma sample was transferred to a 1.5mL microcentrifuge tube and then 20 μL of IS solution (500 ng∙mL^−1^) was spiked. The protein was precipitated by the addition of 280 μL methanol, centrifuged at 11,000×*g* for 30 min at 0 °C after vortexing for 1min, the supernatant was then loaded onto the preconditioned SPE cartridge The cartridge was rinsed by 1mL water, and then eluted with 4mL acetone.

SPE can be performed off-line manually, semi-automated, or on-line. The on-line SPE technique offers speed, high sensitivity by the pre-concentration factor, and low extraction cost per sample, but typically requires the use of program controlled switch valves and column re-configurations. However, the on-line technique can be fully automated. Most on-line SPE approaches use column-switching to couple with the analytical columns. Various column dimensions can be configured for the fast analysis of drug and their metabolites in biological matrix at the nanogram per mililiter level or lower. Wang *et al.* [7] developed a novel on-line SPE column switching HPLC–MS method for the analysis of puerarin in canine and human plasma. Parameters for SPE column switching system (type of the sorbents and percentage of organic modifier) and MS (ionization method and source parameters and ion optics) were optimized for selectivity and sensitivity. The results show that Agilent Zorbax C18 spherical SPE stationary phase could effectively eliminate the interfering materials in plasma and provide a quantitative extraction of puerarin in canine plasma.

### 2.5 Matrix solid-phase dispersion

Matrix solid phase dispersion (MSPD) has found particular application as an analytical process for the preparation, extraction and fractionation of solid, semi-solid and/or highly viscous biological samples. Low sample and solvents consumption, straightforward application, and reduced cost, and its ability to simultaneously perform extraction and clean-up in a single step, are some of its major advantages. Its application to flavonoid analysis was reported only recently. After extraction employing MSPD, the isoflavonoids in *Radix astragali* were determined and identified by the LC–DAD–MS [8]. The MSPD extraction was compared with two conventional extraction methods, ultrasonic and Soxhlet methods. For MSPD C_18_-bonded silica was used and elution was carried out with methanol–water (9:1, v/v). Careful optimization of the eluent composition was needed to prevent co-extraction of interfering matrix components and reduced isoflavonoid yields. It was observed that the best extraction efficiency for formononetin and calycosin was obtained using MSPD, whereas for their glycosides, the conventional Soxhlet method gave better results. However, Soxhlet extraction required 10-fold more sample and solvent and the extraction time was much longer. Ultrasonic extraction gave rather poor results, especially for the aglycones. Taking into account the extraction efficiency and consumption, MSPD extraction is a comparatively better method.

Another study on the phenolic compounds in wines should be mentioned [9]. A novel MSPD extraction method was developed to simultaneously extract 23 phenolic compounds (including quercetin, kaempferol, etc.) from wine samples prior to determination by gas chromatography with mass spectrometric detection in the selected ion monitoring mode. Different parameters of the MSPD technique such as dispersant solid-phase, eluting solvent, and sample ionic strength and pH were optimized. The optimized MSPD procedure requires a small volume of wine (1 mL), commercial silica gel (1.5 g) as dispersant solid-phase and a small volume of ethyl acetate (5 mL) as eluting solvent. Under these conditions, the extraction of the studied compounds was almost complete (mean values of recoveries between 87 and 109%) in a short time (15 min). Moreover, satisfactory standard deviations of repeatability (RSD < 9% in most cases), linear regression coefficients (*r*^2^ > 0.993) and detection limits (< 8 μg/L) confirm the usefulness of the methodology for routine monitoring of the concentration of individual phenolic antioxidants in wines.

The influence of an MSPD-type treatment on the (partial) hydrolysis of glycosides has not been studied in any of the quoted papers. This is an aspect that requires further attention.

### 2.6 Solid-phase micro-extraction

SPME is a modern sampling or sample-preparation method used for isolating and pre-concentrating organic molecules from gaseous, liquid and solid samples. It is highly sensitive and can be used for polar and non-polar analytes with different types of matrix. The mechanism of SPME is similar to that of SPE because SPME is a miniature version of SPE, the only difference being the volume of sorbent. SPME uses a short piece of a fused-silica fiber coated with a polymeric stationary phase placed on a syringe. SPME has also been coupled with LC to analyze non-volatile and/or polar compounds. Using on-line in-tube SPME–LC–DAD UV, Mitani *et al.* [10] determined genistein and daidzein in soybean foods. To improve the robustness of the SPME procedure, an open-tubular fused-silica capillary column instead of a fibre was used, which enables automation and usually provides better performance characteristics than manual techniques. In order to optimize the extraction of these compounds, several in-tube SPME parameters were examined. Optimum extraction conditions for daidzein and genistein were obtained with 20 draw/eject cycles of 40 μL of sample using a porous-layer open-tubular capillary column; the extraction and desorption by the in-tube SPME method were accomplished automatically within 30 min. Analyte recoveries from spiked food were above 97% in all cases.

### 2.7 Supercritical-fluid extraction

Supercritical fluid extraction (SFE) has been applied in the food and medical industries extensively in recent years. The advantages include the ability to perform rapid (often less than 30 min) extractions, to reduce the use of hazardous solvents because carbon dioxide is commonly used as the extraction solvent, and to couple the extraction step with gas, liquid, or supercritical-fluid chromatography. An important aspect of applying SFE to the extraction of active compounds from TCMs is that potential degradation as a result of a lengthy exposure to elevated temperatures and atmospheric oxygen are avoided. Supercritical carbon dioxide extraction was employed to extract flavonoids from *Pueraria lobata* [11]. From response surface plots, pressure, temperature and co-solvent amount exhibited independent and interactive effects on the extraction of flavonoids. The optimal conditions to obtain the highest flavonoid yield were a pressure of 20.04 MPa, a temperature of 50.24 °C and a co-solvent amount of 181.24 mL.

Peng *et al.* [12] used SFE to extract three flavonoids (orotinin, orotinin-5-methyl ether and licoagrochalcone B) from *Patrinia villosa*. The optimization of parameters including pressure, temperature, modifier and sample particle size on yield was carried out using an analytical-scale SFE system. The process was then scaled up by 100 times using a preparative SFE system under the optimized conditions of 25 MPa, 45°C, a sample particle size 40–60 mesh and modified CO_2_ with 20% methanol. The yield of the preparative SFE was 2.82% (crude extract I) and the combined yield of orotinin, orotinin-5-methyl ether and licoagrochalcone B was 0.82 mg/g of dry sample mass.

## 3. Qualitative and Quantitative Analyses

### 3.1 Liquid chromatography

LC analysis of flavonoids is usually carried out in the reversed-phase (RP) mode, on C_18_– or C_8_-bonded silica columns ranging from 100 to 250 mm in length and usually with an internal diameter of 3.9 to 4.6 mm. However, other phases, such as Sephadex, silica and polyamide are also used. Both isocratic and gradient elution are applied for analysis of flavonoids. The mobile phases have been acetonitrile and/or methanol in combination with water containing acetate or formate buffer. Phosphate buffers are less popular than they used to be, mainly because of the dreaded contamination of ion sources when MS detection is used. LC is usually performed at room temperature, but temperatures up to 40°C are sometimes recommended to reduce the time of analysis and because thermostated columns give more repeatable elution times. With the advent of the Ultra performance LC system (UPLC), a new technology is able to operate at high pressure (1000 bar, compared with 400 bar of high-pressure LC), using 1–5 mm i.d. columns with 1–2 μm range particles packing at high flow rates. In this way, the performance of the LC technique is improved: the times for flavonoids analysis are shortened; the resolution and the sensitivity are higher [13,14,15]. For example, Chen *et al.* [15] developed a UPLC method for simultaneous determination of 15 flavonoids in *Epimedium*. The analysis was performed on Waters Acquity UPLC system with an Acquity UPLC BEH C18 column (50 mm × 2.1 mm i.d., 1.7 μm) and gradient elution of 50 mM acetic acid aqueous solution and acetonitrile within 12 min. All calibration curves showed good linearity (R^2^ > 0.9997) within test ranges. The LOD and LOQ were lower than 0.13 and 0.52 ng on column, respectively.

All flavonoid aglycones contain at least one aromatic ring and, consequently, efficiently absorb UV light. The first maximum, which is found in the 240–285 nm range, is due to the A-ring and the second maximum, which is in the 300–550 nm range, to the substitution pattern and conjugation of the C-ring. Simple substituents such as methyl, methoxy and non-dissociated hydroxyl groups generally effect only minor changes in the position of the absorption maxima. Already several decades ago, UV spectrophotometry was still a popular technique to detect and quantify flavonoid aglycones. More recently, diode-array detectors (DAD) give much more information about the full spectra of these compounds, which may be a great help in their identification. Usually the spectra, collected in the range 200-400 nm, are compared with the spectra of reference compounds by evaluating the degree of overlapping. Today UV detection become the preferred tool in LC-based analyses and, LC with DAD is a fully satisfactory tool in studies dealing with, e.g. screening, quantification of the main aglycones and/or a provisional sub-group classification [16,17,18]. 

In flavonoid analysis, fluorescence detection is not very often used [19], because the number of flavonoids that exhibit native fluorescence is limited. Classes of flavonoids that show native fluorescence include the isoflavones, flavonoids with an OH group in the 3-position, e.g. 3-hydroxyflavone and catechin [*trans*-3,3′,4′,5,7-pentahydroxyflavan], and methoxylated flavones, e.g. 3′,4′,5′-trimethoxyflavone. As excitation and emission spectra usually falls in the UV range, this property is very advantageous to the analyst because fluorescence increases sensitivity and minimizes background interference, thus simplifying sample preparation. When fluorescence detection is used in combination with UV it offers the possibility to discriminate between fluorescent and non-fluorescent co-eluting compounds. To extend the application range of fluorescence detection, derivatization of non-fluorescent flavonols could also be used.

Recently, electrochemical detection of phenolics, based on their redox properties has gained diffusion [20]. Since most flavonoids are electroactive due to the presence of phenolic groups, electrochemical detection can also be used. Although ED is not as sensitive as fluorescence detection, LODs can be quite low. With the recent advances in electrochemical detection, multi-electrode array detection is becoming a powerful tool, compatible with gradient elution, for detecting flavonoids in a wide range of samples.

#### 3.1.1 LC−MS and LC–MS/MS

With the introduction of MS, the coupling of LC and MS has opened the way to extensive, routine analysis of flavonoids. LC-MS combines the efficient separation capability of LC and great power of structural characterization and high sensitivity of MS. Compared with GC-MS, LC-MS can determine polar analytes without the need for prior derivatization. This advantage of LC-MS is particularly attractive when simultaneously analyzing compounds belonging to structurally distinct groups. In most cases single-stage MS is used in combination with UV detection to facilitate the confirmation of the identity of flavonoids in a sample with the help of standards and reference data. For the identification of unknowns, tandem mass spectrometry (MS/MS or MS*^n^*) is used—a technique that deserves more attention. With regard to structural characterization of flavonoids, information can be obtained on (1) structure of aglycone, (2) the types of carbohydrates or other substituents present, (3) the sequence of the glycan part, (4) interglycosidic linkages, and (5) attachment points of the substituents to the aglycone. Depending on the nature of the application, additional information is derived from LC retention behaviour, and UV absorbance – and, occasionally, FLU or ED – characteristics, due comparison being made with standard injections and/or tabulated reference data. In studies on the characterization of unknowns, a wide variety of LC–MS/MS techniques is usually applied next to LC–DAD UV for rapid class identification [21]. Many combinations of tandem MS have been tried, the triple quadrupole (TQ), hybrid quadrupole-time of flight (Q-TOF) and quadrupole ion trap (QIT) mass spectrometers are the most successful examples.

In the LC–MS of flavonoids– as in many other application areas –electrospray ionization (ESI) and atmospheric pressure ionization interfaces, i.e. APCI, are used almost exclusively today. These two interfaces show greater ionization stability, and more sensitivity than other interfaces. Both positive ionization (PI) and negative ionization (NI) are applied. According to most studies, for both APCI and ESI the NI mode provides best sensitivity. However, the PI mode should not be neglected, since useful complementary information is often obtained in studies dealing with the identification of unknowns. Recently, a new ionization method for LC/MS, atmospheric pressure photoionization (APPI), has been introduced which is based on charge transfer to the analytes from dopant molecules (e.g., toluene) that have been ionized using 10 eV photons produced by a vacuum-ultraviolet lamp. Matrix-assisted laser desorption ionization (MALDI) is another soft ionization technique, although MALDI-TOF-MS is well-known as powerful tool for analysis of a wide range of biomolecules, its potential in flavonoids analysis has only been explored recently. An example of this application will be illustrated here with rapid screening of anthocyanins in berry samples by surfactant-mediated matrix-assisted laser desorption/ionization time-of-flight mass spectrometry [22]. The addition of the surfactant led to suppression of matrix ions from both *a*-cyano-4-hydroxycinnamic acid (CHCA) and 2′,4′,6′-trihydroxyacetophenone (THAP). It was observed that CHCA led to a great deal of fragmentation of the sugar moiety from glycosides, whereas THAP produced more intact glycoside molecules, and thus led to better characterization of the flavonoids in a berry sample. 

##### 3.1.1.1 LC−MS analysis of flavonoids in plants

During the last years, a large number of different papers concerning the methods for identifying and quantifying flavonoids in plants by tandem mass spectrometry have been published. Table 1 describes relevant information on recent LC−MS analysis of flavonoids in plants; a representative selection is discussed below.

**Table 1:** Recent publications on LC-MS analysis of flavonoids in plants.

Method	Eluents (v/v)	Sample	Flavonoids	Reference
CEC–UV 337nm	pH 2.8 phosphate buffer at 50 mmol L^−1^ containing 50% acetonitrile	*Chamomilla recutita*	11 phenolic compounds including 7 flavonoids	[55]
MEKC–UV 275nm	15 mM borate, 30mM sodium dodecyl sulfate (SDS) and 10% (v/v) ethanol at pH 10.5	*Ixeridium gracile*	Luteolin 7-*O-*glucoside, 2′,4′-dihydroxydihydrochalcone, 2′,4′-dihydroxychalcone, 7-hydroxyflavanone and quercetin 3-*O*-galactoside	[56]
MEKC–UV 214nm	Borate buffer containing SDS at pH 9.0	Various types of wines.	Catechin, naringenin, quercetin, apigenin, kaempferol and myricetin	[57]
MEKC–UV 254nm	50mM borax, 25mM sodium dodecyl sulfate and 30% of acetonitrile(pH 6.75)	*Arnica montana* L.	7 flavonoids and 4 caffeic acid derivatives	[58]
CE–ED	Borate buffer (pH 9.0)	grapefruit peel and juice	Hesperidin, naringin, hesperedin, narigenin and rutin and ascorbic acid	[59]
CZE-AD	β-CD and the mixture of methanol and ethanol as modifiers	Chrysanthemum	Apigenin, luteolin, kaempferol, quercetin, (+)-catechin and (−)-epicatechin	[60]
CZE–UV 270nm	50mM borate buffer (pH 10.0)	*Epimedium*	Icariin, epimedin A, epimedin B and epimedin C	[61]
CE–UV 210nm	pH 9.0 with 30mM borate as buffer containing 8% (v/v) acetonitrile	*Lamiophlomis rotata*	Luteolin-7-*O*-glucoside, isorhamnetin, apigenin, luteolin and quercetin	[62]
CEC–UV 260nm	10 mM ammonium formate, pH 3.0.	*Adinandra nitida*	Epicatechin, camellianin A, rhoifolin camellianin B, apigenin.	[63]

Wang *et al.* [23] reported a HPLC-DAD-ESI-MS^n^ method to identify and characterize the flavonoids in a Chinese formulated preparation, Longdan Xiegan Decoction (LXD). In total, fifty-one flavonoids (27 flavones, 10 flavanones, 7 chalcones, 5 flavonols and 2 isoflavones) were characterized. Eighteen compounds among them including a newly detected flavonoid, naringin, from the ingredient herbs, were unambiguously determined by comparing the retention times (t_R_), UV spectral data and mass fragmentation behaviors with those of the reference compounds. Another thirty-three compounds were tentatively identified by referencing to the reported data of their UV and MS spectra. The ESI-MS/MS fragmentation behavior of flavones (OMe-substituted, *O*-glycosides, *C*-glycosides), chalcones, flavonols and their appropriate characteristic pathways were proposed. In negative ion ESI-MS all the flavonoids yielded prominent [M–H]^−^ ions in the first order mass spectra. Fragmentation with a loss of mass of 15Da (CH_3_), 18Da (H_2_O), 28 Da (CO), 44 Da (CO_2_), 56Da (2CO) and the residues of glucose and glucuronic acid observed in the MS/MS spectra were useful for aiding the structural identification of the flavonoids investigated.

The fragmentation behavior of six flavonoid *O*-diglycosides from genus *Citrus* was investigated using ion trap mass spectrometry in negative ESI with loop injection [25]. For the flavonoid *O*-rutinosides, [*M*−H−308]^−^ ion was typically observed in the MS^2^ spectrum, suggesting the loss of a rutinose. The fragmentation patterns of flavonoid *O*-neohesperidosides were more complicated in comparison with their rutinoside analogues. A major difference was found in the [*M*−H−120]^−^ ion in the MS^2^ spectrum, which was a common feature of all the flavonoid *O*-neohesperidosides. The previous literature for naringin located the loss of 120 Da to the glycan part, whereas the present study for naringin had shown that the [M−H−120]^−^ ion was produced by a retro-Diels-Alder reaction in ring C, and this fragmentation pattern was confirmed by the accurate mass measurement using an orthogonal time-of-flight mass spectrometer. Combined HPLC and DAD, the established approach to the structural identification of flavonoid *O*-diglycosides by ion trap mass spectrometry was applied to the analysis of extracts of two Chinese medicines derived from genus *Citrus*, namely *Fructus aurantii* and *F. aurantii immaturus*. According to the HPLC retention behavior, the diagnostic UV spectra and the molecular structural information provided by MS*^n^* spectra, 13 flavonoid *O*-glycosides in *F. aurantii* and 12 flavonoid *O*-glycosides in *F. a. immaturus* were identified rapidly.

Jin *et al.* [26] developed a screening method based on target molecular weights to characterize the flavonoid glycosides in the flower of *Carthamus tinctorius* L. The screening tables of aglycone and glycan were designed, respectively, in order to select and combine freely. Seventy-seven flavonoid glycosides were screened out finally, and their structures were characterized by tandem mass spectrometric method in both positive and negative ion modes. The glycosylation mode, aglycone, sequence and/or the interglycosidic linkages of the glycan portion and glycosylation position were elucidated by the fragmentation rule in the MS. Numerous compounds were discovered for the first time as much as we known, especially those minor components. Unfortunately, some flavonoid skeleton such as phenylchromone and anthocyanidins and some substituent groups such as the isopropenyl were not included in the screening table.

LC-DAD-ESI/MS was used to identify 23 flavonoids in the extract of Mexican oregano (Lippia graveolens H.B.K.), a spice and herb, used in the USA and Mexico [30]. The identification of luteolin-7-*O*-glucoside, apigenin 7-*O*-glucoside, phloridzin, taxifolin, eriodictyol, scutellarein, luteolin, quercetin, naringenin, pinocembrin and galangin was confirmed by direct comparison with standards. Identification of 6-hydroxyluteolin, two 6-hydroxyluteolin 7-*O*-glycosides, three pentahydroxy- flavanone hexosides, scutellarein 7-*O*-hexoside, 3-hydroxyphloretin hexoside, and three other flavones, was made by detailed analysis of their UV and mass spectral data. The identification of the flavonoid glycosides was further confirmed through detection of their aglycones following hydrolysis of the samples. The concentration of the identified flavonoids in three samples was also estimated. 

To contribute to the quality control of Daqingye and unlocking the secret of its pharmacological activities, a novel method based on UPLC-PDA-ESI-MS/MS was developed for the qualitative and quantitative analysis of flavonoids in its leaves [31]. The separation was carried out on an Acquity UPLC BEH C_18_ column with 0.1% formic acid and methanol as the mobile phase under gradient conditions. Eight flavone *C*-glycosides were identified and their mass spectrometric fragmentation patterns were studied. Among them, the fragmentation pathways of three flavone 6-*C*-diglycosides with the rare 1→3 interglycosidic linkage were investigated for the first time, three characteristic fragment ions [M-H-90]^−^, [M-H-180]^−^, and [M-H-270]^−^ were found in their MS/MS spectra. In addition, a quantitative analytical method for six flavone *C*-glycosides in Daqingye by UPLC-ESI-MS/MS was established and applied for the determination of commercial Daqingye samples from different resources.

Zhang *et al.* [33] described a HPLC-DAD-ESI-MS method for the separation and characterization of flavonoids in *Sophora flavescens* Ait. Based on the chromatographic separation of most flavonoids present in *S. flavescens* Ait., a total of 24 flavonoids were identified. Furthermore, fragmentation pathways in positive ions mode of 24 flavonoid compounds of types of flavanone, flavanonol, flavonol, chalcone, isoflavone, isoflavanone and ptercocarpane were summarized. Some common features, such as CH_3_·, H_2_O, CO, CO_2_, C_3_O_2_ and C_2_H_2_O losses, together with Retro-Diels–Alder fragmentations were observed in the prenylated flavonoids in *S. flavescens* Ait. The loss of the lanandulyl chain was their characteristic fragmentation, which might help deducing the structure of unknown flavonoid compounds. The study provided an approach to rapidly characterize bioactive constituents in *S. flavescens* Ait.

##### 3.1.1.2 *In vivo* studies by LC−MS

The extent and the form of absorption of flavonoids, after oral administration, are importantly unsolved problems in studying their potential effects. A series of reviews on the absorption, metabolism, and bioactivity of flavonoids have been published. Glucuronidation, sulfation and methylation were the main metabolic pathways of flavonoids. Depending on the structure, flavonoid glycosides undergo collision-induced cleavage of the *O*-glycosidic bond producing deprotonated aglycone product ions. Based on the MS fragmentation characteristics, flavonoid metabolites can be supposed. HPLC combined with ESI and APCI interfaces were both used for the quantitative analysis of flavonoids and their metabolites in biofluids. They were quantified directly or determined after enzymatic hydrolysis with authentic standards. The detection by ESI or APCI was carried out in the positive or negative ion mode—the data could be collected in SIM, SRM or MRM mode. Many papers have been published on LC–MS analysis of flavonoids and their metabolites in plasma, urine, bile or feces in recent years [34,35,36,37,38,39,40,41,42,43]. An overview of the LC–MS methods for flavonoids analysis is presented in Table 2, a representative selection is also discussed below.

**Table 2:** Recent publications on *in vivo* analysis by LC-MS.

Flavonoids	Samples	Extraction techniques	Mass analyzer	Scan mode	Reference
Calycosin-7-*O*-β-d-glycoside, Ononin and (*6R*,*10R*)-9,10-dimethoxypterocarpan-3-*O*-β-D-glycoside	rat plasma	SPE	TQ	ESI(+) SRM	[34]
Isorhamnetin, quercetin and kaempferol	rat plasma	LLE	TQ	ESI(+) MRM	[35]
Apigenin and its metabolite, luteolin	rat plasma	PPT	TQ	ESI(−) MRM	[36]
Puerarin	canine and human plasma	SPE	TQ	ESI(−) SRM	[9]
Daidzein, genistein, glycitein, dihydrogenistein, dihydrodaidzein and *O*-desmethylangolensin	human urine	LLE	Single-Q	APCI(−) SIM	[37]
Baicalein, baicalin, oroxylin A and wogonin	rat plasma	LLE	TQ	ESI(+) MRM	[38]
Icariin and its two major metabolites, icariside I and icariside II	rat plasma.	LLE	TQ	ESI(−) MRM	[39]
I3,II8-biapigenin	mouse and rat plasma, brain	SPE	TQ	ESI(−) MRM	[40]
Homoeriodictyol-7-*O*-β-d-glycoside and its active metabolite homoeriodictyol	rat tissues and urine.	LLE	Single-Q	APCI(−) SIM	[41]
Liquiritin apioside, liquiritin, liquiritigenin, isoliquiritin apioside, isoliquiritin, and isoliquiritigenin	rat plasma	LLE	TQ	ESI(−) SIM	[42]

As most of the studies with biapigenin were *in vitro* and there was little information on its bioavailability and distribution in tissues, including brain, and metabolism in animal models after administration of the authentic compound or biflavone-containing herb products. A HPLC–MS/MS method was developed to investigate the pharmacokinetics of I3, II8-biapigen, the major biflavone in *H. perforatum* extracts [40]. The procedure includes solid-phase extraction and separation on an XTerra MS C18. This study has shown for the first time that biapigenin was present in mouse and rat plasma after a standardized *Hypericum perforatum* extract. It was not detected in brain (<5 ng g^−1^), suggesting poor brain-to-blood permeability. Biapigenin concentrations were measurable in mice after intraperitoneal biapigenin (10 mg kg^-1^), but the dosage amounted to about 2% of the equivalent systemic exposure after correction for the contribution from residual blood.

The development of a LC–MS/MS–based method capable of simultaneously quantifying multiple active licorice flavonoids (including liquiritin apioside, liquiritin, liquiritigenin, isoliquiritin apioside, isoliquiritin, and isoliquiritigenin) in plasma was reported [42]. Electrospray ionization was used to efficiently generate precursor deprotonated molecules of all the analytes and the [M–H]^-^ ions were used to produce characteristic product ions for MS/MS analysis. It was found that inclusion of a very low concentration of HCOONH_4_ (0.01%) in the LC mobile phase dramatically improved the detection limit for the tested flavonoids and decreased the interference by matrix effects, which have been referred to as “LC-electrolyte effects.” Liquid–liquid extraction with ethyl acetate was effective for isolation of all the analytes and resulted in the lowest matrix effects of several tested sample cleanup methods. This bioanalytical method showed good linearity between 0.32 ng/mL and 1 μg/mL analyte in 50 μL plasma samples. The accuracy and precision at different analyte concentrations varied from 85 to 110% and from 0.8 to 8.8%, respectively. 

There is little doubt that, in the near future, LC with tandem MS detection will continue to play a dominant role in flavonoid analysis. Next to excellent sensitivity, this technique can provide structural information based on various C-ring cleavages (including RDA fragmentation), and loss of small fragments. It will be an aspect of interest to determine more clearly than has been done until now, to which extent such fragmentation pathways can be used as ‘indicators’ for the presence of specific flavonoids [21]. Generally speaking, attention will, and should, be shifted from analytical method development to more in-depth, and more application-oriented studies, for example in work on the unraveling of metabolic pathways in the human body with the highly interesting anti-oxidant properties of flavonoids as a key issue.

#### 3.1.2 LC–NMR

NMR spectroscopy is a powerful technique for structure elucidation of organic molecules. Therefore, the coupling of LC and NMR could lead to the complete assignment and structure determination of analytes. LC–NMR has become an important technique for the biomedical, pharmaceutical, environmental, food and natural products analysis, as well as for the identification of drug metabolites. In most flavonoid analysis by LC–NMR, the stop-flow mode is the method of choice if more detailed structural analysis is desired, or the sample amount is not enough for on-flow NMR measurement; scan times varied between 1 h and several days per chromatographic peak. As regards real-life applications, a combination of stopped-flow LC/DAD/SPE/NMR and LC/UV/(ESI)MS was used to identify the constituents in *Hypericum perforatum* extract [44]. For the on-line NMR detection, the analytes eluted from column were trapped one by one onto separate SPE cartridges, and hereafter transported into the NMR flow-cell. LC/DAD/SPE/NMR and LC/UV/MS allowed the characterization of constituents of Greek H. perforatum, mainly flavonoids (quercetin, quercitrin, isoquercitrin, hyperoside, astilbin, miquelianin, I3, II8-biapigenin), naphtodianthrones (hypericin, pseudohypericin, protohypericin, protopseudohypericin), phloroglucinols (hyperforin, adhyperforin), and phenolic acids (chlorogenic acid, 3-O-coumaroylquinic acid). 

LC–DAD/UV–SPE–NMR was used in combination with on-line radical scavenging detection for the identification of radical scavenging compounds in extracts of *Rhaponticum* *carthamoides* [45]. A combination of on-line recorded ^1^H NMR spectra, MS/MS fragmentation and exact mass data were used to determine basic structures and elemental composition, while HMBC experiments were performed off-line to determine the sugar-linking type, after trapping the compounds of interest up to three times on separate SPE cartridges and combining the eluates. Without any prior off-line chromatographic steps, five flavonoid-β-glucopyranosides were identified, of which two had a 6′′-*O*-acetyl group.

Despite the promising performance of this hyphenated technique, there is only a small number of publications on LC–NMR for flavonoid analysis every year. Currently, much effort is devoted to the development of micro- or even nano-LC–NMR. This is an attractive development since expensive deuterated solvents, which are required to suppress the eluent background signals in ^1^H NMR, can now be used more easily. Much attention is also paid to probe design, to reduce the problem of poor sensitivity.

### 3.2 Gas chromatography

The advantages of gas chromatography (GC) clearly lie in its high sensitivity of detection for almost all the volatile chemical compounds or non-volatile compounds readily derivatized. Current separation technology uses fused silica capillary columns for GC to achieve high resolution. However, GC-based methods are labor-intensive because derivatization – in most cases directed at the formation of trimethylsilylether (TMS) derivatives – is unavoidable to increase the volatility of the flavonoids and to improve their thermal stability. It should be noted that for flavonoids with more than one hydroxyl substituent, methylation may yield several derivatives, which makes quantification some difficult. Typically in GC, flavonoids are hydrolyzed and converted into their TMS derivatives, injected onto a non-polar column in the split or splitless mode and separated with a linear 30–90 min temperature programme up to 300°C. *N*,*O*-bis-(trimethylsilyl)-trifluoroacetamide (BSTFA) and *N*-(*tert*-butyl- dimethylsilyl)-*N-*methyltrifluoroacetamide (TBDMS) are the most commonly used derivatizing agents, and EI–MS in the selected ion monitoring mode with a source temperature of up to 250 °C is often used for detection. The molecular ion, [M+H]^+^, and fragments formed by the loss of CH_3_ and/or CO and retro-Diels–Alder (RDA) reactions are typically used for detection [21]. GC in the identification of aglycones as silylated derivatives completed by mass selective detection can be regarded as fairly acceptable in the identification of phenolics. Table 3 illustrates the recent interest in GC for flavonoid analysis; and a representative selection is discussed below.

Due to the similarity of the flavonoids of *Kaempferia parviflora* in terms of chemical structure and polarity, K. Sutthanut *et al.* [46] developed an HPLC coupled with UV–vis detection and a GC-FID without derivatization method for simultaneous identification and quantitation of 11 flavonoid constituents in *Kaempferia parviflora*. Limits of detection ranged from a low of 0.1 ppm to a high of 1.0 ppm and the limits of quantitation were a low of 0.5 ppm. In comparison of the HPLC assay with the GC assay without derivatization, the latter was found to be more simple and efficient, and facilitate the simultaneous detection and determination of 11 flavonoids within a reasonable time.

Shen *et al.* [47] reported for the first time a rapid and sensitive GC–MS method to quantify icaritin and desmethylicaritin simultaneously in human serum samples. The serum samples were extracted with ethyl acetate and then derivatized with BSTFA in pyridine (4:1). With genistein as internal standard, calibration curves with good linearity (*R*^2^ > 0.99) within the concentration range of 0.15–10 nM in the selective ion monitoring mode were obtained. Since the method requires only small quantities of sera, does not need isotopically-labeled standards, and can be performed on standard equipment; it can be routinely used for pharmacokinetic studies on *Epimedium*.

**Table 3:** Typical applications of GC to flavonoids analysis.

Method	Sample	Flavonoids	Derivatization	Reference
GC-FID	*Kaempferia parviflora*	11 flavonoids		[46]
GC–MS	Human serum	Icaritin and desmethylicaritin	BSTFA	[47]
GC–MS	*Citrus fruits*	Anthocyanidins and flavanones	TMS and TMS (oximes)	[48]
GC–MS	*Carica papaya* L. *leaf*	7 phenolic compounds including kaempferol and quercetin	BSTFA+TMCS	[49]
GC-(EI)-MS/MS LC-ESI(+))-MS/MS	*Iris germanica*	Unknown derivatives of isoflavonoids	TMS	[50]
LC, GC, LC-MS	*Amelanchier alnifolia* Nutt.	Cyanidin- and Quercetin-Derived Flavonoids		[51]
GC–MS	Greek aromatic plants	Flavonoids and phenolic acids	BSTFA+TMCS	[52]
GC–MS	Wines	23 phenolic Compounds including quercetin, kaempferol,flavonone	BSFTA	[53]
GC–MS	Various herb extracts	Naringenin, galangin, kaempferol, luteolin	In-vial TMS derivz. + extrn.	[54]

The fragmentation patterns and quantitation possibilities of three anthocyanidins (pelargonidin, cyanidin, malvidin), one flavonol (quercetin), two flavones (apigenin, luteolin) and two flavanones (naringenin, hesperetin) have been investigated as trimethylsilyl and as trimethylsilyl (oxime) derivatives by GC-MS [48]. Results proved that anthocyanidins and flavanones form trimethylsilyl (oximes), while flavonol and flavones provide simple trimethylsilyl derivatives. In all cases, characteristic fragments of high masses are formed proper for quantitation purposes. Hydrolysis conditions for naringin, hesperidin and rutin have been optimized, resulting in the quantitative release of naringenin, hesperetin and quercetin together with their corresponding saccharides. These basic studies made possible the identification and quantification of the flavonoid, carboxylic-/amino acid and sugar constituents of citrus fruit juices and albedos, without any extraction/enrichment procedure.

It is obvious that, for the analysis of flavonoids, GC has not been as popular as LC. For such studies, derivatization is needed (and several derivatives may be formed for one analyte), even in HT–GC. They clearly do not outweigh the rapidity of direct LC–MS (/MS) procedures and the possibility to easily screen samples for target analytes as well as unknowns. This, however, does not rule out the usefulness of GC and its outstanding separation capabilities. More efficient in-vial derivatization and the low LODs of SIM mode MS detection are interesting advantages for GC analysis of flavonoids [21]. 

### 3.3 Capillary electrophoresis

CE is a relatively novel technique, which has developed rapidly and matured since its introduction. It has been a powerful tool and widely applied for the analysis of pharmaceuticals, and has been used for flavonoid analysis in the last ten years. Compared with conventional chromatographic methods, CE has many valuable advantages, i.e. excellent separation efficiency, high resolution, short analysis time, being easy to automate, and low solvent and sample consumption.

Most recent papers on the CE analysis of flavonoids are in the field of natural product research, including the analysis of plants, herbs and other plant-derived products. Among the different modes in CE separation, capillary zone electrophoresis (CZE), micellar electrokinetic chromatography (MEKC) and capillary electrochromatography (CEC) are the most widely used, with, typically, a phosphate, ammonium formate or borate buffer or, voltages of 10–30 kV, capillaries of 50–100 μm I.D. and 10–50 nl injection volumes. To date, the detection is usually performed with UV because of its simplicity, convenience and availability, but also electrochemistry detector (ED), fluorescence, amperometric detector (AD) and MS detectors have been successfully applied for CE analysis of flavonoids.

From the previous reviews on flavonoid analysis, only limited attention is devoted to CE and most of the cited references are for pre-2005 publications. For reasons of complementarity, we will pay attention primarily to the 2005–2008 literature [55,56,57,58,59,60,61,62,63,64,65,66,67,68,69]. The focus will be on the analytical procedures and the application of the CE modes for analysis of flavonoids; a representative selection of the papers published in the last 4 years is summarized in Table 4.

Ganzera *et al.* [58] developed and validated a micellar electrokinetic capillary chromatography (MEKC) method for the simultaneous determination of flavonoids and phenolic acids in *Arnica Montana*. By using an electrolyte solution containing 50 mM borax, 25 mM sodium dodecyl sulfate and 30% of acetonitrile, the separation of seven flavonoids and four caffeic acid derivatives was feasible in less than 20 min. The optimized system was validated for repeatability (σ_rel_ ≤4.4%), precision (inter-day σ_rel_ ≤8.13%, intra-day σ_rel_ ≤4.32%), accuracy (recovery rates from 96.8 to 102.4%), sensitivity (limit of detection (LOD) ≤4.5 μg mL^-1^) and linearity (*R*^2^ ≥0.9996), and then successfully applied to assay several plant samples.

CE is sometimes still very difficult to perfectly separate some complicated system, to solve this problem, the addition of modifier is an effective solution. Zhang, *et al*. [60] used capillary zone electrophoresis for simultaneous determination of flavonoids in chrysanthemum. The suitable running buffer modifiers were explored to simultaneously separate and detect six typical flavonoids (apigenin, luteolin, kaempferol, quercetin, (+)-catechin and (−)-epicatechin) which are the main active ingredients in chrysanthemum by capillary zone electrophoresis with amperometric detection (CZE-AD). It was found that when β-cyclodextrin (β-CD) and the mixture of methanol and ethanol were used as running buffer modifiers, a baseline separation of the six analytes could be accomplished in less than 20 min and the detection limits were as low as 10^-7^ or 10^-8^ g mL^-1^. 

**Table 4:** Representative application of CE to the analysis of flavonoids.

Method	Eluents (v/v)	Sample	Flavonoids	Reference
CEC–UV 337 nm	pH 2.8 phosphate buffer at 50 mmol L^−1^ containing 50% acetonitrile	*Chamomilla recutita*	11 phenolic compounds including 7 flavonoids	[55]
MEKC–UV 275 nm	15 mM borate, 30 mM sodium dodecyl sulfate (SDS) and 10% (v/v) ethanol at pH 10.5	*Ixeridium gracile*	Luteolin 7-*O*glucoside, 2′,4′-dihydroxydihydrochalcone, 2′,4′-dihydroxychalcone, 7-hydroxyflavanone and quercetin 3-*O*-galactoside	[56]
MEKC–UV 214 nm	Borate buffer containing SDS at pH 9.0	Various types of wines.	Catechin, naringenin, quercetin, apigenin, kaempferol and myricetin	[57]
MEKC–UV 254 nm	50 mM borax, 25 mM sodium dodecyl sulfate and 30% of acetonitrile (pH 6.75)	*Arnica montana* L.	7 flavonoids and 4 caffeic acid derivatives	[58]
CE–ED	Borate buffer (pH 9.0)	grapefruit peel and juice	Hesperidin, naringin, hesperedin, narigenin and rutin and ascorbic acid	[59]
CZE–AD	β-CD and the mixture of methanol and ethanol as modifiers	Chrysanthemum	Apigenin, luteolin, kaempferol, quercetin, (+)-catechin and (−)-epicatechin	[60]
CZE–UV 270 nm	50 mM borate buffer (pH 10.0)	*Epimedium*	Icariin, epimedin A, epimedin B and epimedin C	[61]
CE–UV 210 nm	pH 9.0 with 30 mM borate as buffer containing 8% (v/v) acetonitrile	*Lamiophlomis rotata*	Luteolin-7-*O*-glucoside, isorhamnetin, apigenin, luteolin and quercetin	[62]
CEC–UV 260 nm	10 mM ammonium formate, pH 3.0.	*Adinandra nitida*	Epicatechin, camellianin A, rhoifolin camellianin B, apigenin.	[63]

Several CEC procedures are published for the analysis of flavonoids. Separation is carried out with packed, monolithic, coated or fused-silica capillaries. To control the quality of Epimedium and its medical preparations [61], a CEC method was developed for the simultaneous determination of seven flavonoids, including hexandraside E, kaempferol-3-O-rhamnoside, hexandraside F, icariin, epimedin A, B, and C, in Epimedium using baicalein as internal standard (IS). The influence of relevant parameters such as buffer concentration, pH, and proportion of ACN was investigated and optimized. Baseline separation was obtained using a Hypersil C_18_ capillary (3 μm, 100 μm/25 cm) with a mixture of 20 mM phosphate buffer (pH 4.0)/ACN (70:30 v/v) as mobile phase running at 30 kV and 25 °C in 20 min. 

CE–MS has also been used for the analysis of flavonoids [68] and anthocyanins [69]. In the CE–ESI–MS for characterization of the methanolic extract of hops [68], the following CE–ESI-MS conditions were finally selected: running buffer 80 mM ammonium acetate/ ammonium hydroxide, pH 10.5, voltage 25 kV, 10 s injection time, sheath liquid 2-propanol/water 60:40 with 0.1% v/v TEA delivered at a flow rate of 0.28 mL/h, a drying gas flow rate at 4 L/min and at 300 °C, and nebulizing gas pressure 5 psi, and MS analyses were carried out using a compound stability of 25%. The proposed method permits the identification of hop polyphenols (flavonoids glycosides and chalcones), bitter acids (a-acids and b-acids), and their oxidation products. 

With the number of CE studies on analysis of flavonoids rapidly increasing, CE has turned out to be a useful complementary tool for qualitative and quantitative analysis of flavonoids. CE coupled various detectors greatly enhance the capability of this technique. In future, the complementarity of CE separation to LC, and their robust interfacing with MS are promising aspects to explore to analyze flavonoids.

## 4. Conclusions

The huge number of publications appearing on the analysis of flavonoids over the past four decades testifies to the significance of the subject. In this article, we reviewed recent progress made in several areas including sample preparation, separation and detection for flavonoids. The results from many applications cited in this article have demonstrated that innovative chromatography technologies are re-shaping the way of analysis for flavonoids. These techniques have been reported to be accurate and reproducible and in the case of GC–MS, LC–DAD-MS and CE-MS. For the sake of high throughput analysis, we have recently observed the emergence of UPLC coupled to mass spectrometry as an alternative to traditional high-performance liquid chromatography techniques.

Today, we may conclude that – primarily as a result of recent, and still on-going developments in tandem-MS detection – the analytical tools for the reliable detection, identification and quantification of even low concentrations of flavonoids, i.e. aglycones and conjugates, are available. Further developments may be expected with regards to miniaturization, that is, the coupling of micro- and/or nano-LC, and also CE, to tandem- MS and NMR instruments: This should facilitate the analysis of minute samples, and help to create better operating conditions for NMR detection. The future for chromatographic analysis of flavonoids is multi-methods, and several different analytes are expected to be determined simultaneously in a single run. This is feasible by the use of LC–MS-NMR, which makes qualitative and quantitative determination of compounds or their metabolites possible even at very low concentration.

## References

[1] Davies D. D., Stumpf P.K., Conn E.E. (1981). Secondary Plant Products. The Biochemistry of Plants: A Comprehensive Treatise.

[2] Formica J.V., Regelson W. (1995). Review of the Biology of Quercetin and Related Bioflavonoids. Food Chem. Toxicol..

[3] Jiang Y., Li P., Li S.P., Wang Y.T., Tu P.F. (2007). Optimization of pressurized liquid extraction of five major flavanoids from *Lysimachia clethroide*. J. Pharm. Biomed. Anal..

[4] Zhang Y., Li S.H., Wu X.W. (2008). Pressurized liquid extraction of flavonoids from *Houttuynia cordata* Thunb. Sep. Pur. Tech..

[5] Chen L.G., Jin H.Y., Ding L., Zhang H.R., Li J., Qu C.L., Zhang H.Q. (2008). Dynamic microwave-assisted extraction of flavonoids from *Herba Epimedii*. Sep. Pur. Tech..

[6] Zhang X., Sun Y.G., Cheng M.C., Wang Y.Q., Xiao H.B. (2007). Simultaneous quantification of three isoflavonoid glycosides in rabbit plasma after oral administration of *Astragalus mongholicus* extract by high-performance liquid chromatography coupled with electrospray ionization tandem mass spectrometry. Anal. Chim. Acta.

[7] Wang Q.Q, Li X.S., Dai S.J. (2008). Quantification of puerarin in plasma by on-line solid-phase extraction column switching liquid chromatography–tandem mass spectrometry and its applications to a pharmacokinetic study. J. Chromatogr. B.

[8] Xiao H.B., Krucker M., Albert K., Liang X.M. (2004). Determination and identification of isoflavonoids in Radix astragali by matrix solid-phase dispersion extraction and high-performance liquid chromatography with photodiode array and mass spectrometric detection. J. Chromatogr. A.

[9] Minuti L., Pellegrino R. (2008). Determination of phenolic compounds in wines by novel matrix solid-phase dispersion extraction and gas chromatography/mass spectrometry. J. Chromatogr. A.

[10] Mitani K., Narimatsu S., Kataoka H. (2003). Determination of daidzein and genistein in soybean foods by automated on-line in-tube solid-phase microextraction coupled to high-performance liquid chromatography. J. Chromatogr. A.

[11] Wang L.Z., Yang B., Du X.Q., Yi C. (2008). Optimisation of supercritical fluid extraction of flavonoids from Pueraria lobata. Food Chem..

[12] Peng J.Y., Fan G.R., Chai Y.F., Wu Y.T. (2006). Efficient new method for extraction and isolation of three flavonoids from *Patrinia villosa* Juss. by supercritical fluid extraction and high-speed counter-current chromatography. J. Chromatogr. A.

[13] Qi L.W., Wen X.D., Cao J., Li C.Y., Li P., Yi L., Wang Y.X., Cheng X.L., Ge X.X. (2008). Rapid and sensitive screening and characterization of phenolic acids, phthalides, saponins and isoflavonoids in Danggui Buxue Tang by rapid resolution liquid chromatography-diode array detection coupled with time-of-flight mass spectrometry. Rapid Commun. Mass Spectrom..

[14] Qi L.W., Cao J., Li P., Yu Q.T., Wen X.D., Wang Y.X., Li C.Y., Bao K.D., Ge X.X., Cheng X.L. (2008). Qualitative and quantitative analysis of Radix Astragali Products using rapid resolution liquid chromatography-diode array detection coupled with time-of-flight mass spectrometry with dynamic adjustment of fragmentor voltage. J. Chromatogr. A.

[15] Chen X.J., Ji H., Zhang Q.W., Tu P.F., Wang Y.T., Guo B.L., Li S.P. (2008). A rapid method for simultaneous determination of 15 flavonoids in Epimedium using pressurized liquid extraction and ultra-performance liquid chromatography. J. Pharm. Biomed. Anal..

[16] Chen J., Song Y., Li P. (2007). Capillary high-performance liquid chromatography and mass spectrometry for simultaneous determination of major flavonoids, iridoid glucosides and saponins in Flos Lonicerae. J. Chromatogr. A.

[17] Yu Q.T., Qi L.W., Li P., Yi L., Zhao J., Bi Z.M. (2007). Determination of seventeen main flavonoids and saponins in the medicinal plant Huang-qi (Radix Astragali) by HPLC-DAD-ELSD. J. Sep. Sci..

[18] Chen C.Y., Qi L.W., Li H.J., Li P., Yi L., Ma H.L., Tang D. (2007). Simultaneous determination of iridoids, phenolic acids, flavonoids, and saponins in Flos Lonicerae and Flos Lonicerae Japonicae by HPLC-DAD-ELSD coupled with principal component analysis. J. Sep. Sci..

[19] Paulke A., Schubert-Zsilavecz M., Wurglics M. (2006). Determination of St. John’s wort flavonoid-metabolites in rat brain through high performance liquid chromatography coupled with fluorescence detection. J. Chromatogr. B.

[20] Klejdus B., Vacek J., Adam V. (2004). Determination of isoflavones in soybean food and human urine using liquid chromatography with electrochemical detection. J. Chromatogr. B.

[21] Rijke E., de Out P., Niessen W.M.A., Ariese F. (2006). Analytical separation and detection methods for flavonoids. J. Chromatogr. A.

[22] Grant D.C., Helleur R.J. (2008). Rapid screening of anthocyanins in berry samples by surfactant-mediated matrix-assisted laser desorption/ionization time-of-flight mass spectrometry. Rapid Commun. Mass Spectrom..

[23] Wang Y., Yang L., He Y. Q., Wang C.H., Welbeck E.W. (2008). Characterization of fifty-one flavonoids in a Chinese herbal prescription Longdan Xiegan Decoction by high-performance liquid chromatography coupled to electrospray ionization tandem mass spectrometry and photodiode array detection. Rapid Commun. Mass Spectrom..

[24] Petsalo A., Jalonen J., Tolonen A. (2006). Identification of flavonoids of *Rhodiola rosea* by liquid chromatography-tandem mass spectrometry. J. Chromatogr. A.

[25] Shi P.Y., He Q., Song Y., Qu H.B., Cheng Y.Y. (2007). Characterization and identification of isomeric flavonoid *O*-diglycosides from genus *Citrus* in negative electrospray ionization by ion trap mass spectrometry and time-of-flight mass spectrometry. Anal. Chim. Acta.

[26] Jin Y., Xiao Y.S., Zhang F.F. (2008). Systematic screening and characterization of flavonoid glycosides in *Carthamus tinctorius* L. by liquid chromatography/UV diode-array detection/electrospray ionization tandem mass spectrometry. J. Pharm. Biomed. Anal..

[27] Tiberti L.A., Yariwake J.H. (2007). Identification of flavonols in leaves of *Maytenus ilicifolia* and *M. aquifolium* (Celastraceae) by LC/UV/MS analysis. J. Chromatogr. B.

[28] Su J., Fu P., Shen Y.H., Zhang C. (2008). Simultaneous analysis of flavonoids from *Hypericum japonicum* Thunb.ex Murray (Hypericaceae) by HPLC-DAD–ESI/MS. J. Pharm. Biomed. Anal..

[29] Zhang X., Xiao H.B., Xue X.Y. (2007). Simultaneous characterization of isoflavonoids and astragalosides in two Astragalus species by high performance liquid chromatography coupled with atmospheric pressure chemical ionization tandem mass spectrometry. J. Sep. Sci..

[30] Lin L.Z., Mukhopadhyay S., Robbins R.J. (2007). Identification and quantification of flavonoids of Mexican oregano (Lippia graveolens) by LC-DAD-ESI/MS analysis. J. Food Compos. Anal..

[31] Deng X.Y., Gao G.H., Zheng S.N., Li F.M. (2008). Qualitative and quantitative analysis of flavonoids in the leaves of *Isatis indigatica* Fort. by ultra-performance liquid chromatography with PDA and electrospray ionization tandem mass spectrometry detection. J. Pharm. Biomed. Anal..

[32] Lai J.P., Lim Y.H., Su J. (2007). Identification and characterization of major flavonoids and caffeoylquinic acids in three *Compositae* plants by LC/DAD-APCI/MS. J. Chromatogr. B.

[33] Zhang L., Xua L., Xiao S.S. (2007). Characterization of flavonoids in the extract of *Sophora flavescens* Ait. By high-performance liquid chromatography coupled with diode-array detector and electrospray ionization mass spectrometry. J. Pharm. Biomed. Anal..

[34] Zhang X., Sun Y.G., Cheng M.C., Wang Y.Q., Xiao H.B. (2007). Simultaneous quantification of three isoflavonoid glycosides in rabbit plasma after oral administration of *Astragalus mongholicus* extract by high-performance liquid chromatography coupled with electrospray ionization tandem mass spectrometry. Anal. Chim. Acta.

[35] Lan K., Jiang X.H., He J.L. (2007). Quantitative determination of isorhamnetin, quercetin and kaempferol in rat plasma by liquid chromatography with electrospray ionization tandem mass spectrometry and its application to the pharmacokinetic study of isorhamnetin. Rapid Commun. Mass Spectrom..

[36] Wan L.L., Guo C., Yua Q., Li Y., Wang X.W., Wang X.L., Chen C.L. (2007). Quantitative determination of apigenin and its metabolism in rat plasma after intravenous bolus administration by HPLC coupled with tandem mass spectrometry. J. Chromatogr. B.

[37] Chen L.J., Zhao X., Fang L.Y., Games D. E. (2007). Quantitative determination of acetyl glucoside isoflavones and their metabolites in human urine using combinedliquid chromatography–mass spectrometry. J. Chromatogr. A.

[38] Kim Y.H., Jeong D.W., Paek I.B. (2006). Liquid chromatography with tandem mass spectrometry for the simultaneous determination of baicalein, baicalin, oroxylin A and wogonin in rat plasma. J. Chromatogr. B.

[39] Xu W., Zhang Y.P., Yang M. (2007). LC–MS/MS method for the simultaneous determination of icariin and its major metabolites in rat plasma. J. Pharm. Biome. Anal..

[40] Colovic M., Caccia S. (2008). Liquid chromatography–tandem mass spectrometry of I3, II8-biapigenin, the major biflavone in *Hypericum perforatum* extracts. J. Chromatogr. B.

[41] Zhao Y.L., Yu Z.G., Zhang L.C., Zhou D.D., Chen X.H., Bi K.S. (2007). Simultaneous determination of homoeriodictyol-7-*O*-β-d-Glccopyranoside and its metabolite homoeriodictyol in rat tissues and urine by liquid chromatography–mass spectrometry. J. Pharm. Biomed. Anal..

[42] Li L., Liang S.P., Du F.F., Li C. (2007). Simultaneous Quantification of Multiple Licorice Flavonoids in Rat Plasma. J. Am. Soc. Mass. Spectrom..

[43] Tsao R., Deng Z. (2004). Separation procedures for naturally occurring antioxidant phytochemicals. J. Chromatogr. B.

[44] Tatsis E.C., Boeren S., Exarchou V. (2007). Identification of the major constituents of Hypericum perforatum by LC/SPE/NMR and/or LC/MS. Phytochemistry.

[45] Miliauskas G., van Beek, de W. T.A., Venskutonis P.R.P., Sudholter E.J.R. (2005). Identification of radical scavenging compounds in Rhaponticum carthamoides by means of LC-DAD-SPE-NMR. J. Nat. Prod..

[46] Sutthanut K., Sripanidkulchai B., Yenjai C., Jay M. (2007). Simultaneous identification and quantitation of 11 flavonoid constituents in *Kaempferia parviflora* by gas chromatography. J. Chromatogr. A.

[47] Shen P., Wong S.P., Yong E.L. (2007). Sensitive and rapid method to quantify icaritin and desmethylicaritin in human serum using gas chromatography–mass spectrometry. J. Chromatogr. B.

[48] Füzfai Zs., Molnár-Perl I. (2007). Gas chromatographic–mass spectrometric fragmentation study of flavonoids as their trimethylsilyl derivatives: Analysis of flavonoids, sugars, carboxylic and amino acids in model systems and in citrus fruits. J. Chromatogr. A.

[49] Canini A., Alesiani D., Arcangelo G..D., Tagliatesta P. (2007). Gas chromatography–mass spectrometry analysis of phenolic compounds from Carica papaya L. Leaf. J. Food Compos. Anal..

[50] Maul R., Schebb N., Kulling H.S.E. (2008). Application of LC and GC hyphenated with mass spectrometry as tool for characterization of unknown derivatives of isoflavonoids. Anal. Bioanal. Chem..

[51] Ozga J.A., Saeed A., Wismer W., Reinecke D.M. (2007). Characterization of Cyanidin- and Quercetin-Derived Flavonoids and Other Phenolics in Mature Saskatoon Fruits (*Amelanchier alnifolia* Nutt.). J. Agric. Food Chem..

[52] Proestos C., Boziaris I.S., Nychas G.-J.E., Komaitis M. (2006). Analysis of flavonoids and phenolic acids in Greek aromatic plants: Investigation of their antioxidant capacity and antimicrobial activity. Food Chem..

[53] Minuti L., Pellegrino R. (2008). Determination of phenolic compounds in wines by novel matrix solid-phase dispersion extraction and gas chromatography/mass spectrometry. J. Chromatogr. A.

[54] Fiamegos Y.C., Nanos C.G., Vervoort J., Stalikas C.D. (2004). Analytical procedure for the in-vial derivatization- extraction of phenolic acids and flavonoids in methanolic and aqueous plant extracts followed by gas chromatography with mass-selective detection. J. Chromatogr. A.

[55] Fonseca F.N., Tavares M.F.M., Horváth C. (2007). Capillary electrochromatography of selected phenolic compounds of *Chamomilla recutita*. J. Chromatogr. A.

[56] Zhang Y., Chen J., Ma X.M., Shi Y.P. (2007). Simultaneous determination of flavonoids in *Ixeridium gracile* by micellar electrokinetic chromatography. J. Pharm. Biomed. Anal..

[57] Sun Y., Fang N., Chen D.D.Y., Kingsley K.D. (2008). Determination of potentially anti-carcinogenic flavonoids in wines by micellar electrokinetic chromatography. Food Chem..

[58] Ganzera M., Egger C., Zidorn C., Stuppner H. (2008). Quantitative analysis of flavonoids and phenolic acids in *Arnica montana* L. by micellar electrokinetic capillary chromatography. analytica chimica acta..

[59] Wu T., Guan Y.Q., Ye J.N. (2007). Determination of flavonoids and ascorbic acid in grapefruit peel and juice by capillary electrophoresis with electrochemical detection. Food Chem..

[60] Zhang S., Dong S.Q., Chi L.Z. (2008). Simultaneous determination of flavonoids in chrysanthemum by capillary zone electrophoresis with running buffer modifiers. Talanta.

[61] Chen X.J., Ji H., Wang Y.T., Li S.P. (2008). Simultaneous determination of seven flavonoids in Epimedium using pressurized liquid extraction and capillary electrochromatography. J. Sep. Sci..

[62] Luo M.N., Lu H.W., Ma H.Q. (2007). Separation and determination of flavonoids in *Lamiophlomis rotata* by capillary electrophoresis using borate as electrolyte. J. Pharm. Biomed. Anal..

[63] Zhang L.Y., Zhang J., Wang H. (2005). Analysis of flavonoids in leaves of Adinandra nitida by capillary electrochromatography on monolithic columns with stepwise gradient elution. J. Sep. Sci..

[64] Chen J., Li S.L., Li P., Song Y., Chai X.Y., Ma D.Y. (2005). Qualitative and quantitative analysis of active flavonoids in *Flos Lonicerae* by capillary zone electrophoresis coupled with solid-phase extraction. J. Sep. Sci..

[65] Song Y., Li P., Wang D., Cheng Y.Y. (2008). Micellar Electrokinetic Chromatography for the Quantitative Analysis of Flavonoids in the Radix of Astragalus membranaceus var. mongholicus. Planta Med..

[66] Cheung R.H.F., Marriott P.J., Small D.M. (2007). CE methods applied to the analysis of micronutrient in foods. Electrophoresis.

[67] Ehala S., Vaher M., Kaljurand M. (2005). Characterization of phenolic profiles of northern european berries by capillary electrophoresis and determination of their antioxidant activuty. J. Agric. Food Chem..

[68] Arráez-Román D., Cortacero-Ramírez S. (2006). Characterization of the methanolic extract of hops using capillary electrophoresiselectrospray ionization-mass spectrometry. Electrophoresis.

[69] Segura-Carretero A., Puertas-Mejía M.A. (2008). Selective extraction, separation, and identification of anthocyanins from *Hibiscus sabdariffa* L. using solid phase extraction-capillary electrophoresis-mass spectrometry (time-of-flight /ion trap). Electrophoresis.

